# Effects of functionalization and silane modification of hexagonal boron nitride on thermal/mechanical/morphological properties of silicon rubber nanocomposite

**DOI:** 10.1038/s41598-023-39203-5

**Published:** 2023-07-24

**Authors:** Atefe Farahani, Masoud Jamshidi, Masumeh Foroutan

**Affiliations:** 1 Constructional Polymers and Composites Research Lab., School of Chemical, Petroleum and Gas Engineering, Iran University of Science and Technology (IUST), Tehran, Iran; 2 School of Chemistry, College of Science, University of Tehran, Tehran, Iran

**Keywords:** Chemical engineering, Materials for energy and catalysis, Nanoscale materials, Soft materials, Other nanotechnology

## Abstract

Hexagonal boron nitride (h-BN) nanoparticles could induce interesting properties to silicone rubber (SR) but, the weak filler-matrix interfacial interaction causes agglomeration of the nanoparticles and declines the performance of the nanocomposite. In this work, h-BN nanoparticles were surface modified using vinyltrimethoxysilane (VTMS) at different concentrations. Before silane modification, h-BN nanoparticles were hydroxylated using 5 molar sodium hydroxide. The nanoparticles were characterized to assess success of silane grafting. The pure and modified h-BN nanoparticles were applied at 1, 3 and 5 wt% to HTV silicon rubber (SR). The curing, thermal, mechanical and morphological properties and hydrophobicity of the nanocomposites were evaluated. The morphology of the SR nanocomposites was characterized using AFM and FE-SEM analysis. It was found that silane grafting on the h-BN nanoparticles improves crosslink density but declines curing rate index (CRI) of the SR nanocomposite (at 5 wt% loading content) by 0.7 (dN m) and 3.5%, respectively. It also increased water contact angle of the nanocomposites from 97.5° to 107°. The improved nanoparticle-rubber interfacial interactions caused better dispersion of h-BN nanoparticles in SR matrix (at 5 wt%) that enhanced the elongation at break, modulus at 300% and Tg of the SR nanocomposites.

## Introduction

Today polymeric insulators are vastly used as replacement for glassy and porcelain materials because of low weight, simple casting, easy transport/better handling, corrosion and abrasion resistances. On this basis, silicone rubber (SR) is vastly used as insulator in electrical applications^1,2^. However, heat dissipation is the main problem of silicone rubber because of low heat conductivity and thermal stability which is crucial in a thermal interfacial material (TIM) for insulating applications^3,4^. Ceramic fillers like alumina (Al_2_O_3_), aluminum nitrate (AlN) and boron nitride (BN) have been used to improve thermal conductivity and electrical insulation of silicone rubber, hitherto^5,6^. Among all micro and nano fillers used in silicone rubber, boron nitride has unique properties like high thermal conductivity (600 W/m K) as well as breakdown strength, low dielectric loss and permittivity, high thermal stability, low thermal expansion and interesting anisotropic properties because of its layer by layer structure^7–10^.

Although hydrophilic hexagonal boron nitride (h-BN) nanoparticles have great potential to improve silicone rubber properties, but they agglomerate in polymers because of low filer-polymer interfacial interactions^11–13^. This problem mainly affects the dispersion of h-BNs in the polymers which leads to undesirable mechanical and thermal/electrical properties^14^. Surface modification could improve interfacial interactions of h-BNs to polymers^15^. The most challenging part in surface modification of boron nitride nanoparticles is their high chemical stability (with no functional groups on the surface) and lack of bonding sites (i.e. hydroxyl or amine groups) even at the edges^16^. On this basis, an additional step (i.e. functionalization) is necessary before surface modification of h-BNs which is hydroxylation with high concentrated acid or basic solutions. It has been found that after functionalization coupling agents could graft to the surface but mainly at the edges of h-BN^16–18^.

Silane coupling agents as amphiphilic materials with both organic and alkoxy functional groups in one molecule could react with inorganic h-BN after functionalization and improve filler–polymer interfacial interactions^19^.

Silane surface modified h-BNs have been used in different polymers, hitherto. Cheng et al.^20^ showed that chain size of alkoxy side in silane coupling agent have strong effect on thermal conductivity of h-BN. Silanes with smaller organic chaines like vinyl triethoxysilane (VTES) and tetraethyl orthosilicate (TEOS) increased the TC of polyvinyl alcohol (PVA) while longer chains declined it. Wang et al.^21^ confirmed that silanized h-BNs could remain dispersed in paraffin for long times compared to untreated h-BNs. Seyhan et al.^22^ found that silanization of h-BN with vinyl trimethoxy silane could assist the chemical exfoliation of h-BNs that this positive impact on thermal conductivity of polypropylene matrix. Zhong et al.^23^ modified the surface of exfoliated h-BNs with aminopropyltriethoxysilane (KH550) and vinyl trimethoxysilane (KH171). They added the modified h-BNs to silicone rubber (SR). Their results showed that vinyl silane improved the interfacial bonding to rubber matrix and enhanced thermal stability of SR nanocomposite.

In this work, h-BN was functionalized with hydroxyl groups and then modified by vinyltrimethoxysilane (VTMS). The main reason for choosing VTMS was its compatibility to the organic side chains of rubber backbones. After that the pure and modified h-BNs were incorporated into a HTV silicone rubber at three loading contents (i.e. 1, 3, 5 wt%). The influences of pure and modified h-BNs on the thermomechanical, rheological, morphological and hydrophobic properties of SR was evaluated. We tried our best to make a clear and comprehensive investigation on modified h-BN/SR nanocomposites.

## Experimental works

### Materials

The used materials in this study are showed in Table 1.

**Table1:** List of the used materials.

Materials	Company (country)	Description
Hexagonal boron nitride (h-BN)	Pishro Ceramic Mehr (Iran)	Average size: 500 nm–1 μm
Silicone rubber (HTV)	Wacker (Germany)	With dicumyl peroxide (DCP) as curing agent
Sodium hydroxide	Dr Mojalali (Iran)	Flakes (USP)
Vinyl trimethoxy silane	SISIB (china)	Industrial grade
Ammonium hydroxide	Dr Mojalali (Iran)	Concentration: 25%
Absolute ethanol	Pars alcohol (Iran)	Concentration: 99.8%
Ethanol	Pars alcohol (Iran)	Concentration: 70%

### Functionalization of h-BNs

Two gram of h-BN nanoparticles was poured in 5 M sodium hydroxide solution and stirred at 80 °C and 1000 rpm for 12 h. Thereafter, the precipitated part was rinsed with distilled water three times to neutralize the pH. The functionalized powder was dried in vacuum oven at 80 °C for 12 h.

### Surface modification of h-BNs

The functionalized h-BNs were added to absolute ethanol, ammonium hydroxide and water at weight ratio of 1:150:1:1 to prepare a suspension. The mixture was placed in ultrasonic bath at 0 °C and 40 Hz for 30 min. Thereafter, the suspension poured in to a two-necked flask and stirred at rate of 1000 rpm and temperature of the mixture increased to 90 °C. Finally, VTMS was added dropwise to the suspension and the mixture stirred for 4 h to prepare silane grafted h-BNs. The solution was centrifuged for better separation for 10 min at 1500 rpm. The precipitated part was washed three times with ethanol 70% to remove unreacted silane molecules. To complete silane condensation step, the silanized h-BNs were dried in an oven at 80 °C for 12 h^24^.

**Figure 1:**
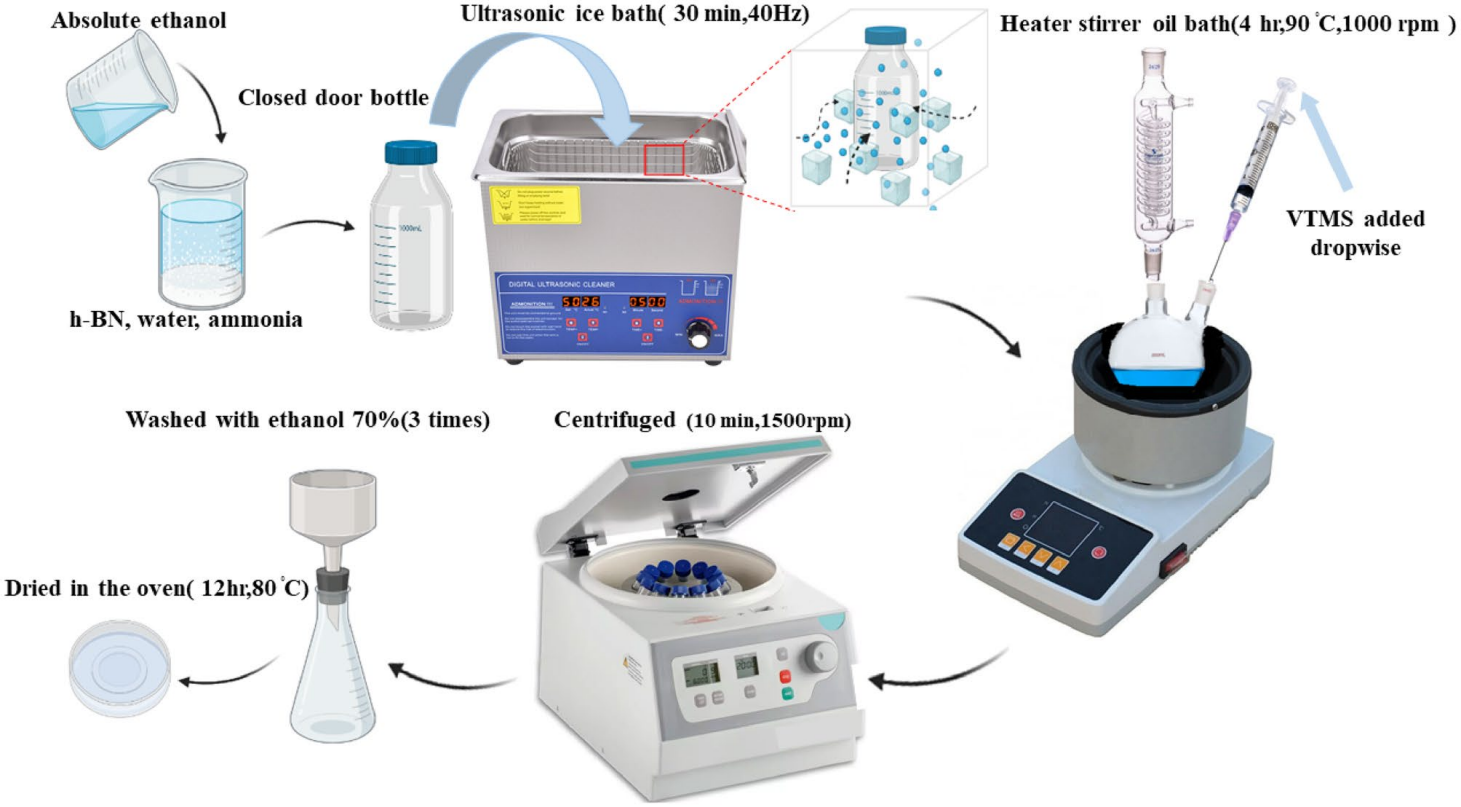
Scheme of silane grafted h-BN preparation method.

To find stoichiometric (i.e. X) content of VTMS for surface modification, firstly the hydroxyl content of the functionalized h-BNs was determined using TGA analysis. The surface modification was performed at different silane concentrations (i.e. 1 X, 5 X and 10 X) to maximize surface grafting. The difference between the weight loss of h-BN and functionalized h-BN (i.e. BN-OH) at the temperature range of 110–600 °C was attributed to degradation of the hydroxyl groups^24^. The hydrolyzed silane molecule has three hydroxyl groups to react with the nanoparticle OH groups, but because of steric hindrance just one hydroxyl group of a silane could bond covalently to the h-BN surface. The following equation was used to calculate the amount of silane coupling agent (SCA) for 1 g of h-BN:

1 $${m}_{SCA}=\frac{{m}_{OH}\times {M}_{SCA}}{{M}_{OH}}$$

where m_SCA_, M_SCA_, m_OH_, M_OH_ are in order the mass of SCA (g), molecular mass of SCA (g/mol), weight of hydroxyl groups per unit gram of nanoparticle and M_OH_ is molecular mass of OH group. Schematic of preparation method of silane grafted h-BNs is illustrated in Fig. 1.

### Preparation of SR nanocomposites

To prepare SR nanocomposites (i.e. at nanoparticles contents of 1, 3 and 5 wt%), the silicon rubber and h-BNs (pure and modified) were blended in an internal mixer (HAAKE mixer) at rolling speed of 80 rpm and room temperature for 10 min. Then, 0.5 g of curing agent (i.e. DCP) was added to the compound and the mixing continued for 2 min. After that, the compounds was mixed by two roll mill (for 10 min) to prepare rubber sheet with thickness of 2 mm. Finally, the rubber compounds were molded and cured via hot-press at 160 °C. The vulcanization parameters of rubber compounds were derived from moving die rheometer (MDR) at 160 °C. Based on the MDR results, the curing of nanocomposites was performed at 160 °C for 6 min. The samples were post cured in an oven at 200 °C for 4 h.

### Characterization

In order to find out the efficiency of silane grafting, different analysis were performed. Firstly, the silane modification of h-BNs was examined by TGA analysis (METTLER TOLEDO, Star^e^ SW14) which was held under nitrogen atmosphere at rate of 10 °C/min in temperature range of 40–600 °C. Fourier transform infrared spectroscopy (FTIR, Perkin Elmer) and X-ray diffraction (XRD, Dron-8) and X-ray photoelectron spectroscopy (XPS) (Thermo Electron Co, Waltham, USA) analysis were performed to characterize the chemical structure, crystallinity and the chemical bonds of pure and silanized h-BNs. Atomic force microscopy (AFM) (Ara Multi mode AFM instrument) and field emission scanning electron microscopy (FE-SEM) (TESCAN MIRA 3 LMU device) were also performed to study morphology of the nanoparticles.

The tensile properties of the SR nanocomposites were evaluated by an Instron Universal Testing Machine according to ASTM D 412^25^. The cure characteristics of the rubbery samples were determined using a moving die rheometer (MDR, A0225-rheo Techpro) at 160 °C according to ASTM D5289^26^. TGA analysis was held to evaluate thermal behavior of the rubbery samples. The analysis was performed between 30 and 800 °C with a heating rate of 10 °C/min in Argon atmosphere. Water contact angle test was held to investigate the influence of VTMS on hydrophobicity of the nanocomposites using a Data Physics instrument (Dino-lite handheld digital microscope) with individual droplet volume of 2 μL. The average contact angle (CA) was determined by three separate measurements at different locations for each sample. The dynamic mechanical thermal analysis (DMTA) was done on all rubbery samples at frequency of 1 Hz, rate of 10 °C/min from − 100 to 100 °C by METTLER TOLEDO DMA1 instrument. Besides, atomic force microscopy (AFM) and field emission scanning electron microscopy (FE-SEM) were used to study morphology of SR nanocomposites.

### Sample coding

The codes of prepared sample are presented in Table 2.

**Table 2:** The codes of prepared samples.

Full name	Code
Hexagonal boron nitride nanosheets	h-BN
Vinyl trimethoxy silane	VTMS
Silanized boron nitride with VTMS (m = 1, 5, 10)	sBN-mX
Silicone rubber-boron nitride nanocomposites (wt% = 1, 3, 5)	R-BN (wt%)
Silicone rubber-silanized boron nitride nanocomposites (wt% = 1, 3, 5)	R-sBN (wt%)

## Results and discussions

### Surface modified h-BNs

#### The optimal silane concentration

**Figure 2:**
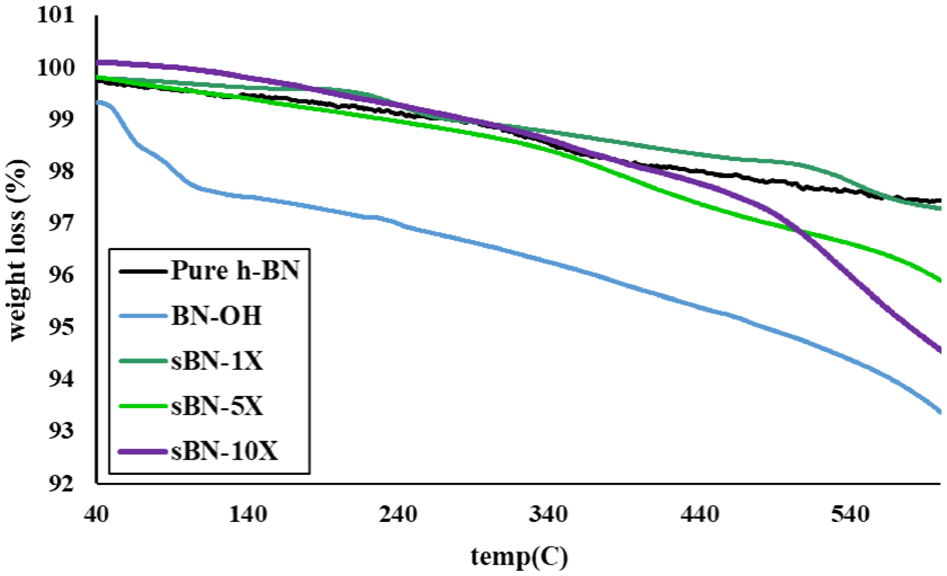
TGA analysis of pure and modified h-BNs.

Figure 2 and Table 3 show results of TGA analysis for pure and modified h-BNs. As previously mentioned, TGA is very helpful to find the necessary amount of silane to react with h-BN surface hydroxyl groups^24^. For pure h-BNs, a slight weight loss (about 2.5%) was observed in temperature range of 40–600 °C. Hydroxylation changed the thermal stability of h-BNs and made two visible weight losses that were related to degradation of absorbed humidity and hydroxyl groups. After silane treatment, the slope and amount of weight loss before 400 °C was closely like to pure h-BNs which showed that most of surface OH groups were consumed during reaction to silane molecules. The weight loss increased in the modified h-BNs between 400 and 600 °C that was attributed to degradation of grafted silanes^27^. In sBN-1 X sample, low silane grafting was occured but undesirable contaminations that produced during production of h-BNs washed out. On this basis, the overall weight loss was even fewer than pure h-BNs. Because of this unexpected result, TGA analysis was repeated, but the same results were achieved again. Besides, the highest weight loss was observed in sBN-10 X sample that was corresponded to its higher silane grafting content. Grafting ratio (Rg) of VTMS on h-BNs was also calculated based on the weight loss at temperature range of 400–600 °C. The highest Rg (%) was observed for sBN-10 X sample. On this basis, this sample was used for further investigations. Detailed information listed in Table 3.

**Table 3:** TGA data of pure and modified h-BNs.

Sample	Weight loss (%)			The amount of silane (g)	Grafting ratio (%)
	40–160 °C	160–400 °C	400–600 °C		
Pure h-BN	0.55	1.29	0.8	–	–
BN-OH	1.61	1.8	2.5	–	–
sBN-1X	0.2	0.7	1.04	0.13	0.24
sBN-5X	0.56	1.52	2.33	0.65	1.53
sBN-10 X	0.3	1.62	3.92	1.3	3.12

#### Chemical characteristics of h-BNs

**Figure 3:**
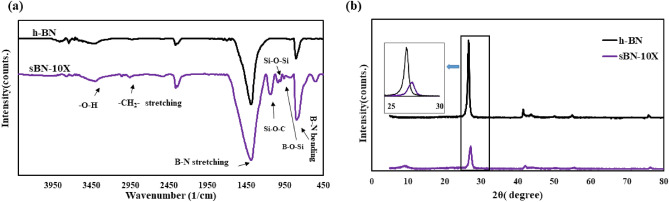
(**a**) FTIR, (**b**) XRD analysis of pure and modified h-BNs.

To characterize the chemical structure of pure and modified h-BNs, FTIR and XPS analysis were performed that results are depicted in Fig. 3. Figure 3a shows FTIR spectrums. The results showed efficient grafting of VTMS on h-BNs. In all spectrums, two main peaks at 1385 and 805.5 cm^−1^ was observed that related to the plane stretching vibration of B-N bond and out of plane optical mode of B–N–B bending vibration, respectively^28^.

The peak at around 2300 cm^−1^ was attributed to the identified transmission peak of CO_2_^29^. However, some researchers corresponded this peak to the reverse reaction that happens during production of h-BN which produces boric acid as a side product^30,31^. The peak located at 1450 cm^-1^ was corresponded to the stretching vibration of B–N–B group that overlapped by bending vibration peak of B–OH (a small shoulder appeared in the sBN-10 X diagram). After silanization, lots of peaks showed up in sBN-10 X spectrum. The first revealing peak at 2954 cm^−1^ was attributed to CH_2_ asymmetric and symmetric stretching vibration. Besides, at 962 cm^−1^, B–O–Si band showed up which was directly an evidence for reaction between hydrolyzed silane molecules and h-BN surface hydroxyl groups. The peaks related to Si–O–C and Si–O–Si stretching vibration were observed at 1006 and 1120 cm^−1^, respectively^32^. In addition, the peak observed at 540 cm^−1^ was corresponded to Si–O rocking vibration^33^. The results confirmed successful grafting of VTMS molecules on h-BN surface.

XRD results were also presented in Fig. 3b. The wide and low intense peak generated around 10° was attributed to the products of self-assembly (i.e. homo-polymerization) of VTMS molecules^12^. Besides, after surface modification the tendency for making crystalline structure reduced and as a result the intensity and position of (002) plane peak which showed up at 26.56° for pure h-BNs changed significantly for sBN-10 X sample (27.18°)^34^. Based on the Debye–Scherrer equation^35^ crystalline size of the samples was calculated. It was found that the crystalline size was altered from 21.6 nm for h-BN to 20.7 nm for sBN-10 X.

**Figure 4:**
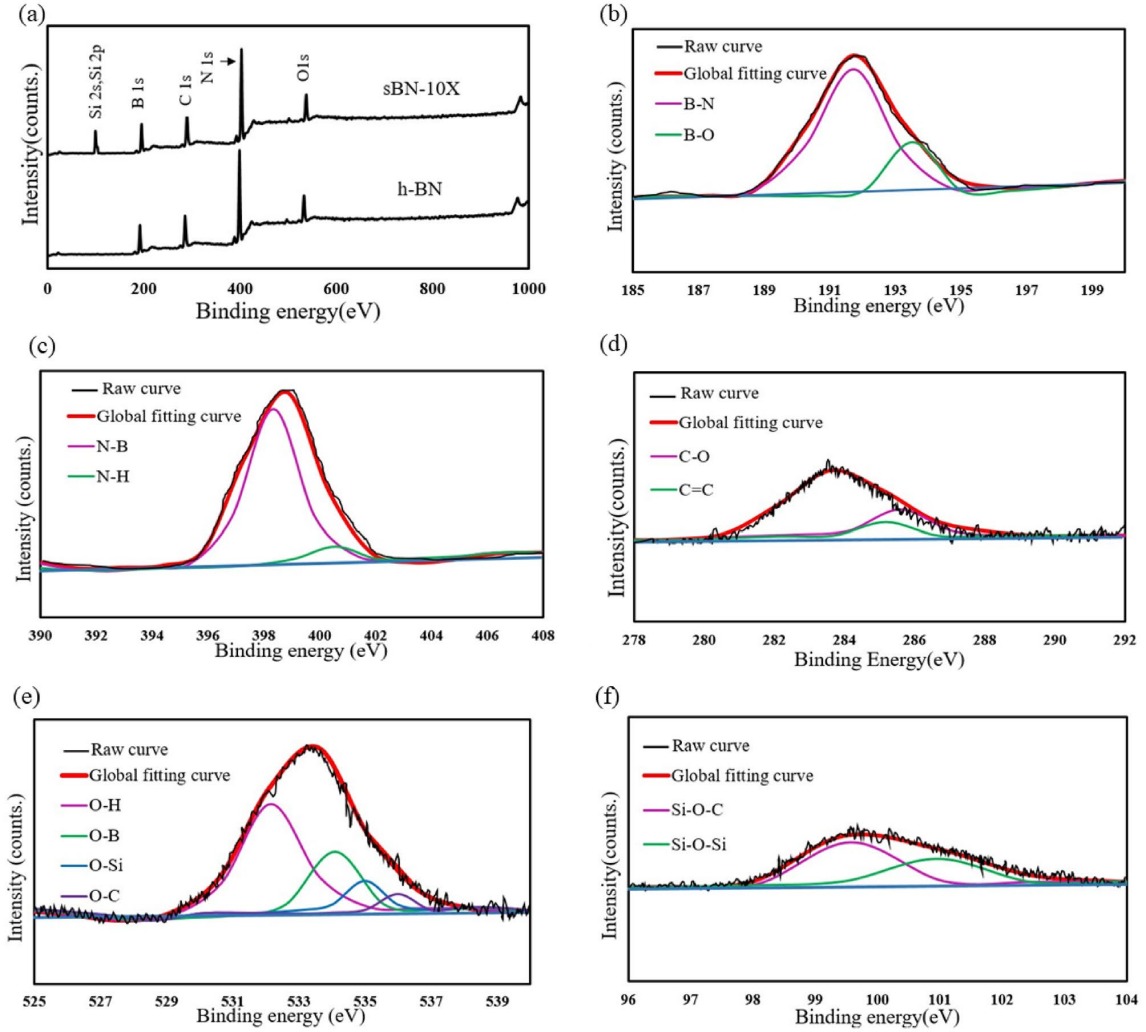
(**a**) XPS spectra for h-BN and sBN-10 X, high resolution XPS spectra for sBN-10 X: (**b**) B 1*s*, (**c**) N 1*s*, (**d**) C 1*s*, (**e**) O 1*s*, (**f**) Si 2*s*, Si 2*p*.

X-ray photoelectron spectroscopy (XPS) was performed on the samples to assess success of silane grafting on h-BNs. Figure 4 showed that the main h-BN peaks related to B 1*s*, N 1*s*, O 1*s* and C 1*s* were exactly the same for both pure and modified samples. However, Si element was detected in modified h-BNs that was corresponded to Si–O–Si group at 101.5 eV and Si–O–C bond at 99.57 eV. Spectra of oxygen element showed the presence of VTMS related bonds and hydroxylation of h-BN surface before silane treatment. In addition, the concentration of carbon element increased after silanization which is another evidence for successful grafting of VTMS on h-BNs^24,34^. Besides, according to molecular dynamic (MD) investigations on surface modified h-BNs, if the grafting agents want to absorb or covalently bond to the surface of h-BN layer, they should break B–N bond or change the electron orbitals of boron element which both of them induce structural distortion^35^. As a result, the reaction of VTMS molecules to h-BNs mainly happens at the edges that this decreases the silane grafting content. Further information of XPS results are listed in Table 4.

**Table 4:** Results of XPS analysis.

Peak	Related bonds (position)	Concentration (%)	
		h-BN	sBN-10 X
B 1*s*	B–N (191.8 eV), B–O (194.1 eV)	5.25	5.21
N 1*s*	N–B (398.3 eV), N–H (400.8 eV)	78.43	77.75
O 1*s*	O–H (532.2 eV), O–B (534.1 eV), O–Si (535 eV), O–C (538.7 eV)	14.83	11.51
C 1*s*	C=C (285.7 eV), C–O (286.3 eV)	1.49	2.68
Si 2*s*	Si–O–C (99.57 eV)	0	1.53
Si 2*p*	Si–O–Si (101.5 eV)	0	1.32

#### Morphological characteristics

**Figure 5:**
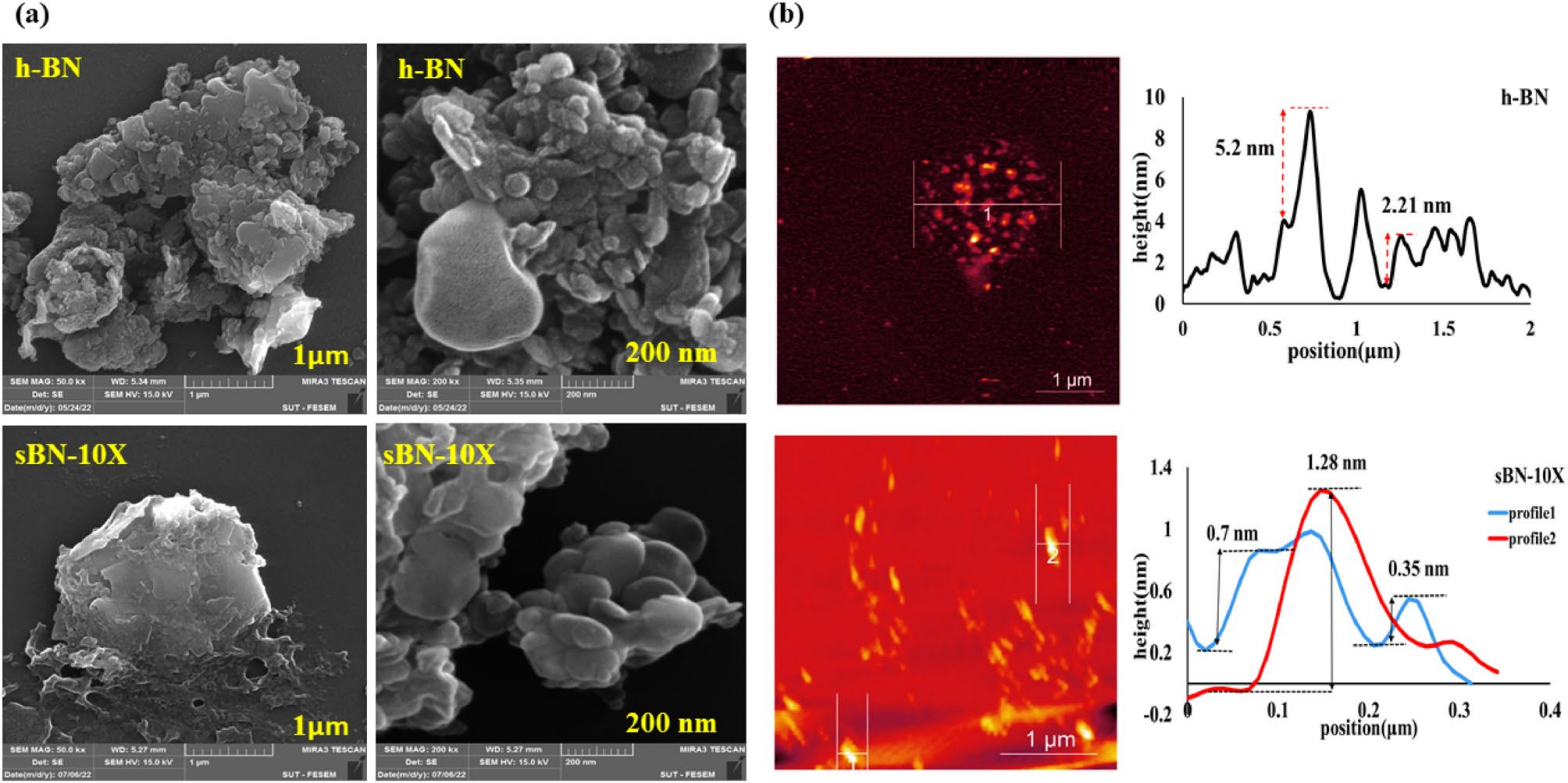
Morphological analysis of pure and modified h-BNs; (**a**) FE-SEM images at 1μm and 200 nm and (**b**) AFM analysis at 1μm.

VTMS has two effects on morphology of the surface of h-BN. It could reduce the particle size of h-BNs because of vinyl sides and their steric repulsion. It could also increase the surface roughness of the h-BNs. Figure 5 depicts results of FE-SEM and AFM analysis of pure and modified h-BNs. FE-SEM pictures from the surface at scales 1 μm and 200 nm showed that the sharpness of edges in h-BNs reduced after surface modification (see Fig. 5a). Besides, the modified h-BNs swelled up because of penetration of silane molecules between the layers and the brighter edges could be a sign for presence of silane molecules^18,36^. The difference of h-BNs agglomerates are clearly seen in the images at scale of μm. This was attributed to the act of vinyl side of VTMS in sBN-10 X sample that properly hindered layer by layer attachments. The images at scale of 200 nm also revealed smaller sized agglomerates in the modified h-BNs that confirmed action of silane coupling agents as exfoliating agent for 2D boron nitride^23^. As a result, VTMS molecules were grafted on h-BNs and at the same time reduced the layers thickness.

The roughness of samples was examined by AFM analysis which results are depicted in Fig. 5b. The images obviously verified lower size of agglomerates in the modified h-BNs compared to the pure h-BNs. An accumulation of little sized agglomerations at the middle of h-BN image revealed high tendency of them to be attached to each other. The role of VTMS is to reduce the size,s increase the nano layers departure and decrement in the agglomerates roughness. The diagrams verified that the roughness of attached layered drastically reduced from around 5 nm in pure h-BN to 0.35 nm for modified h-BN and even more the sharpness of peaks declined because of VTMS coating on h-BNs surface.

### SR nanocomposites

#### Curing properties

**Figure 6:**
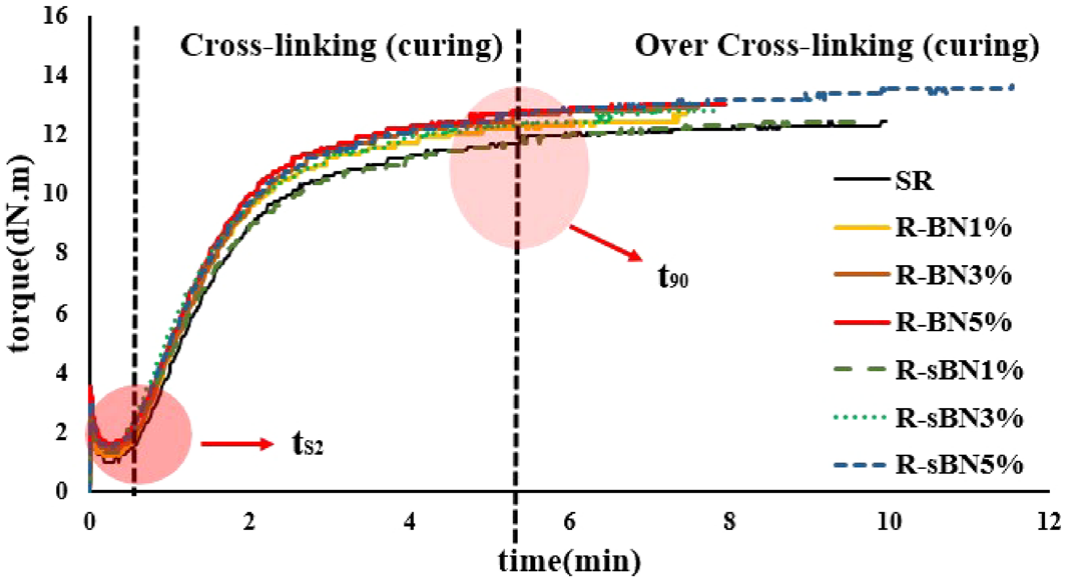
The curing behavior of SR compounds.

Previously it was concluded that among all modified samples sBN-10 X showed higher grafting ratio. As a result, it was used in preparation of SR nanocomposites. The curing results of SR nanocomposites are depicted in Fig. 6 and Table 5 at vulcanization temperature of 160 °C. During an isothermal vulcanization process, three stages happened after each other. The first stage is scorch time which is caused by collaboration of different components of a system to decrease the deformation resistance. Changing the slope of curing diagram shows starting the second stage in which crosslinking happens rapidly. At the third stage, the vulcanized network develops^36^. The plateau pattern in the third region shows the stability of curing process and means that network may no longer change (see Fig. 6)^37,38^.

**Table 5:** Curing properties of the rubber compounds.

Sample	M_H_–M_L_ (dN m)	CRI (%)	t_s2_ (s)	t_90_ (s)
R	11.46	31.545	50	241
R-BN1%	11.47	37.03	48	210
R-BN3%	11.48	37.03	49	211
R-BN5%	11.59	36.23	47	213
R-sBN1%	11.47	35.71	48	216
R-sBN3%	11.71	33	43	225
R-sBN5%	12.21	32.67	46	230

The pure h-BNs with high thermal conductivity acts as thermal zones in SR matrix and transfers heat better. So during curing, the pure and modified h-BN containing nanocomposites showed a slight decrease in the optimum curing time (t_90_) and scorch time (t_s2_) compared to pristine SR sample. The scorch time fell more in the modified h-BN containing nanocomposites compared to the pure h-BN containing samples. However, the optimum curing time slightly declined in the modified nanocomposites due to the surface modification of h-BNs. This silanization improved dispersion of h-BNs in SR matrix and enhanced their heat conduction capacity that decreased rubber curing time. The cure rate index (i.e. CRI) of all nanocomposites also calculated. All the nanocomposites exhibited higher curing rate than pristine SR, but silane grafting hindered the performance of heat conductive nano layers and reduced CRI of the modified samples. The increased M_H_–M_L_ confirms more crosslinking in the modified nanocomposites due to the better dispersion of sBN-10 X in the SR matrix.

#### Thermal stabilities

**Figure 7:**
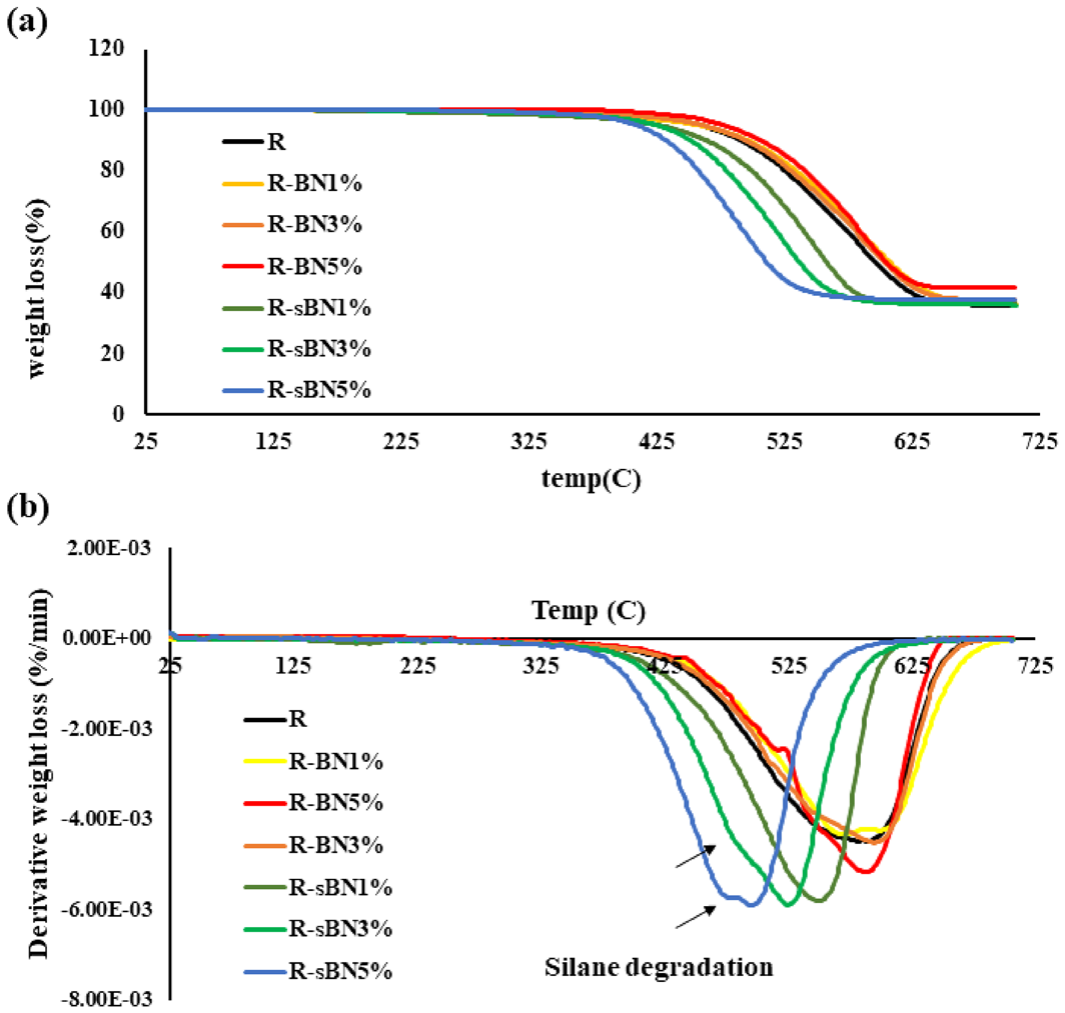
Thermogravimetric analysis of pristine and modified SR nanocomposites; (**a**) TGA and (**b**) DTG curves.

TGA analysis was conducted to find out the thermal performances of nanocomposites. Results are depicted in Fig. 7. According to the curves, pure h-BNs increased the thermal stability of SR and at the end of heating process the residual mass was higher than pristine SR (see Fig. 7a). In contrary, silane grafted h-BNs reduced the thermal stability of silicon rubber. As discussed previously, better dispersion of modified h-BNs in the rubber matrix was the reason for improved heat conduction. When these well dispersed thermal zones with a lower interfacial resistance started to heat up, the degradation process of the matrix in their boundaries speeded up, too. Besides, the defects and voids can also be a reason for reduction in the thermal stability which are caused by incorporation of more silane grafted h-BNs in the SR matrix^39^. In addition, the weight loss content was negligible in R-sBNs under 200 °C which means that the covalent bond happened between VTMS and h-BN and there is no free radical of VTMS in the system^40^. In DTG curves, a small shoulder was observed especially at higher sBN contents at temperature around 400 °C which came from grafted silane degradation. On this basis, it could be assumed that the early degradation process with sharper decomposition rate happened because of silane decomposition and the respective reduction in thermal stability with increase in sBN content could be a sign for that. The results of DTG analysis tabulated in Table 6. As it can be seen, addition of pure h-BNs significantly increased the ash content of SR nanocomposites but modified h-BNs had no effect on residual mass of SR matrix. It was happened because of more agglomerations in SR matrix in pure h-BN containing nanocomposites which hindered the heat conduction and thermal degradation as a result of their high heat capacity^28^. However, modified h-BNs dispersed better in the SR matrix and created better heat conduction paths so the degradation process of rubber continued faster. Grafted silane molecules were decomposed until 700 °C and as a result it became reasonable that the remained mass of R-sBNs reduced.

**Table 6:** TGA and DTG information of nanocomposites.

Samples	Temperature at 5% weight loss (T_5_)	First degradation temperature (T_1max_)	Final degradation temperature (T_2max_)	Ash content at 700 °C (wt%)
R	448	408	665	35.7
R-BN1%	449	382	670	36.17
R-BN3%	450	395	660	37.48
R-BN5%	454	440	660	41.5
R-sBN1%	415	372	600	36.52
R-sBN3%	412	362	590	36.86
R-sBN5%	403	360	570	37.42

#### Morphological properties

**Figure 8:**
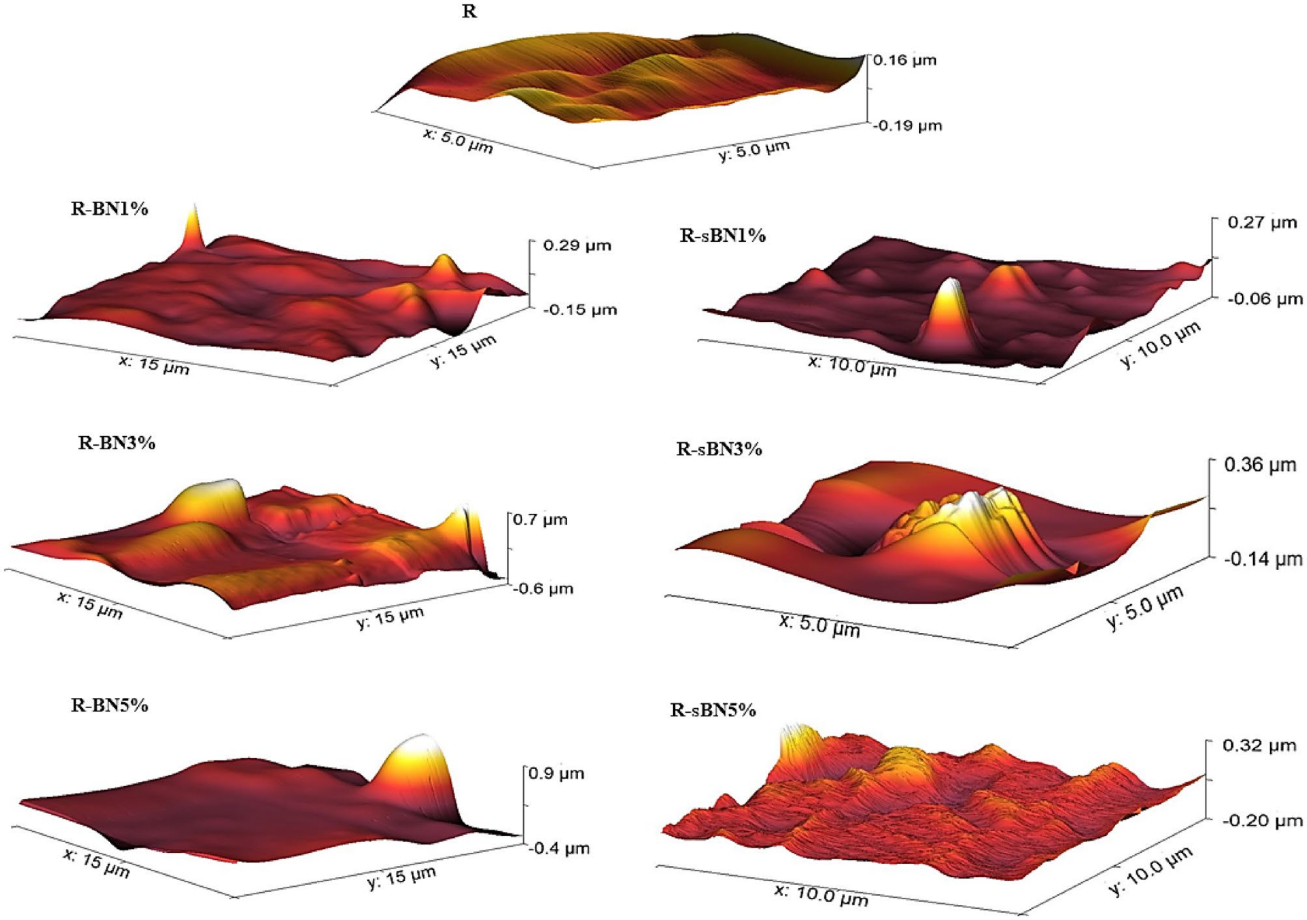
AFM 3D images of SR nanocomposites.

The surface roughness can effectively increate hydrophobicity of the nanocomposites^24^. AFM 3D images of the nanocomposites are presented in Fig. 8. Addition of pure h-BNs to the SR matrix made the smooth surface more rough with some peaks which were bigger at h-BNs higher contents. Besides, weak dispersion of pure h-BNs is obviously seen in these samples. The modified h-BNs showed better dispersion in SR matrix while overall roughness of the surface increased. Because the roughness formula is integral of all peaks on a specified length, so better dispersion spreads peaks better on the surface and increase the overall roughness. The sharpness of the peaks was also altered after silanization. The surface roughness of all samples were put in the Table 7.

**Table 7:** Surface roughness of samples.

Samples	Roughness (nm)
R	4.1
R-BN1%	6.13
R-BN3%	10.88
R-BN5%	25.91
R-sBN1%	63.11
R-sBN3%	80.26
R-sBN5%	96.02

**Figure 9:**
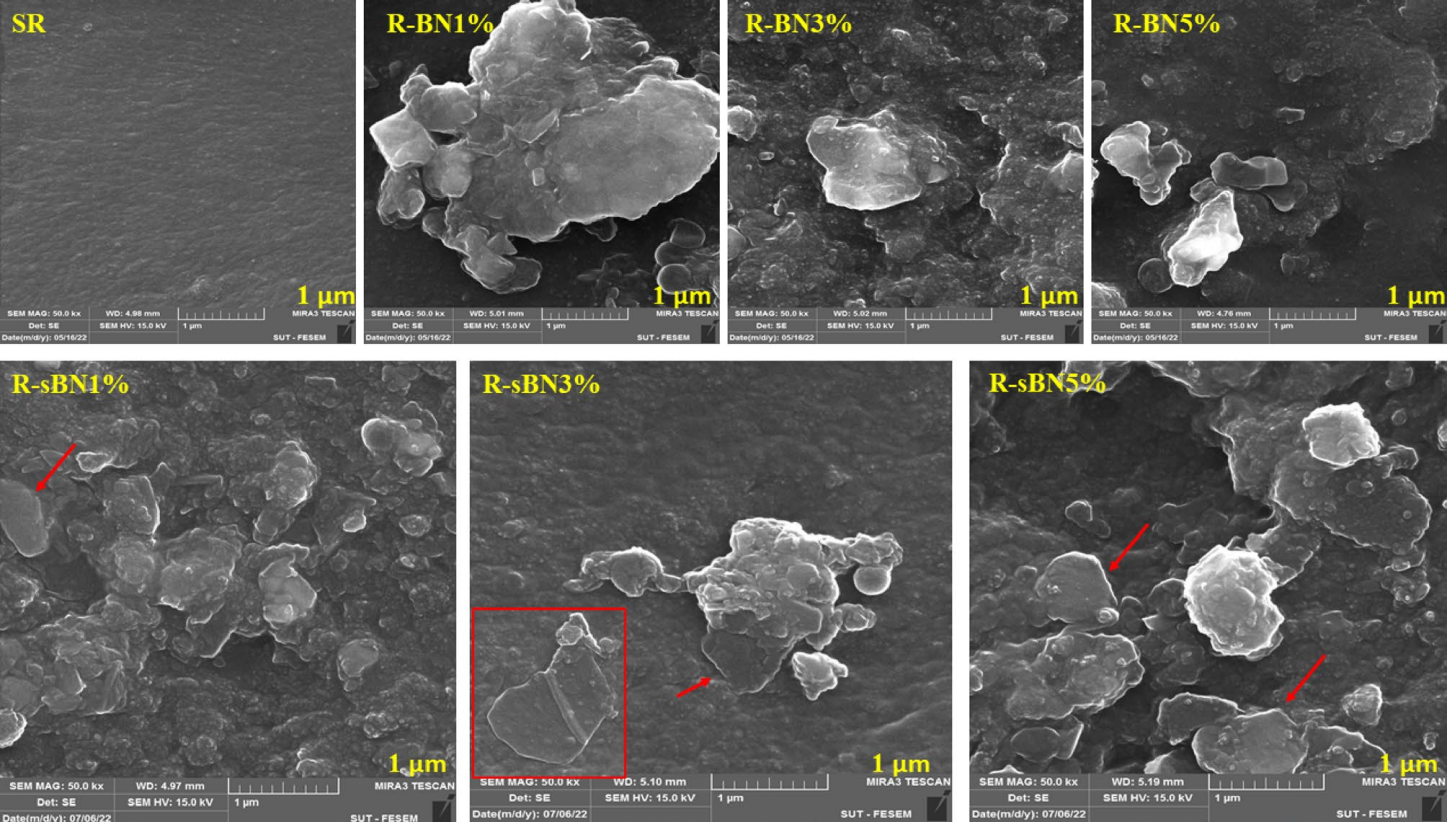
FE-SEM images from surface of the SR nanocomposites.

**Figure 10:**
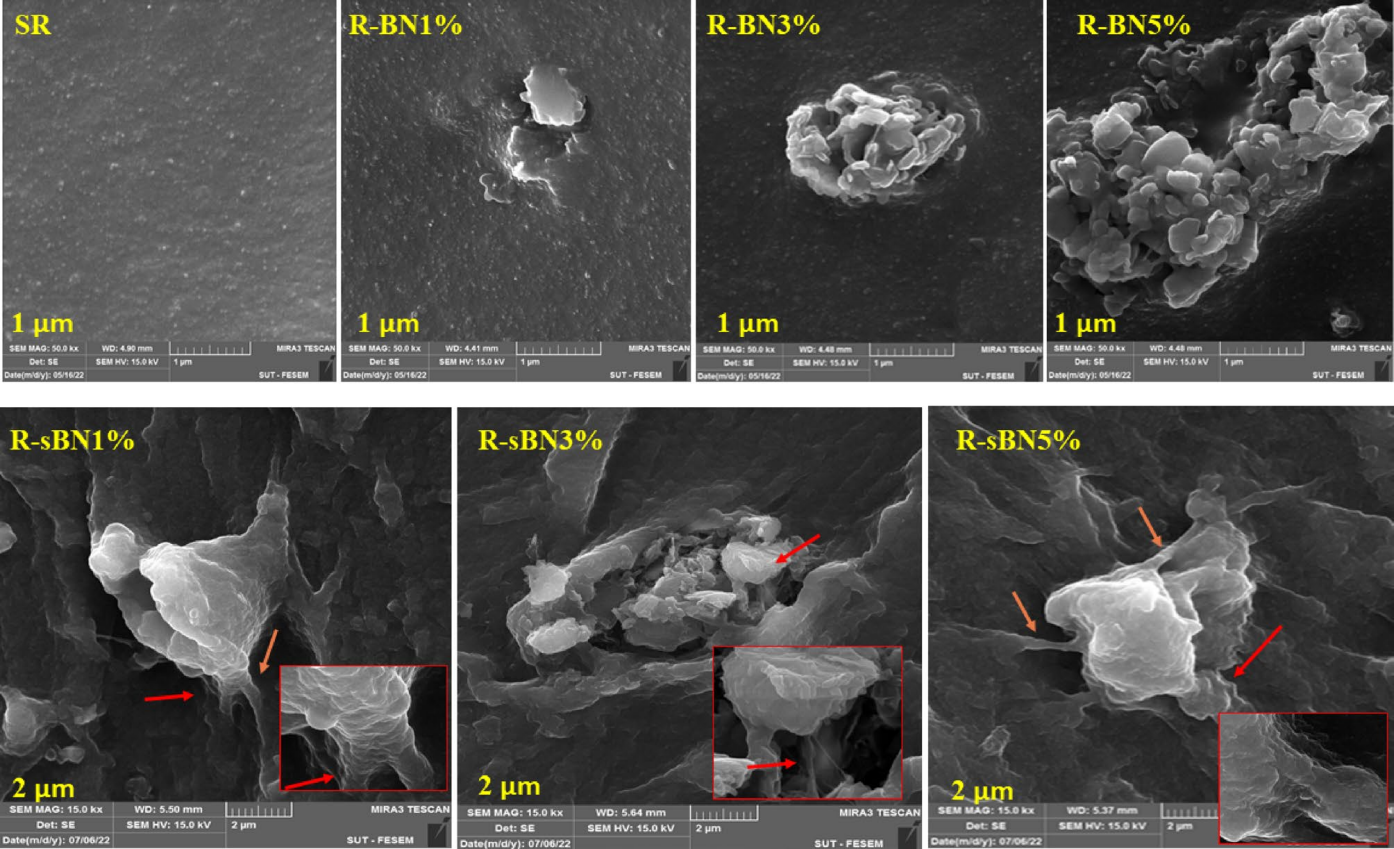
FE-SEM images from fractured surface of the nanocomposites.

FE-SEM images were captured from the surface and fractured surface of the nanocomposites. Results are shown in Figs. 9 and 10. Figure 9 represents the images of the surfaces of nanocomposites at scale of 1 μm. SR surface was completely soft but the surface roughness increased by addition of pure h-BNs due to creation of big agglomerates which were visibly departed from SR matrix. The VTMS grafting caused efficient dispersion of h-BNs in rubber matrix. In modified nanocomposites at all h-BNs contents, intact and large surfaces of nano layers were clearly seen that wetted by rubber matrix. It was found that silane modified h-BNs had less agglomerations than pure ones even at the same roughness^41^.

The performance of VTMS became more visible in images captured from the fractured surface of the nanocomposites. In pure h-BN containing nanocomposites, increasing in the h-BN loading content caused creation of bigger agglomerates (see Fig. 10). Obvious bridges between modified h-BNs and SR matrix was seen especially at the edges of the nano layers that confirmed the grafting of VTMS on these areas (i.e. because of weaker B–N bonds in those areas and formation of more OH groups during functionalization)^35^. Besides the morphology of the agglomerates altered from discrete into sticky nano sheets which is more significant at the corner of R-sBNs images.

#### Water contact angle

**Figure 11:**
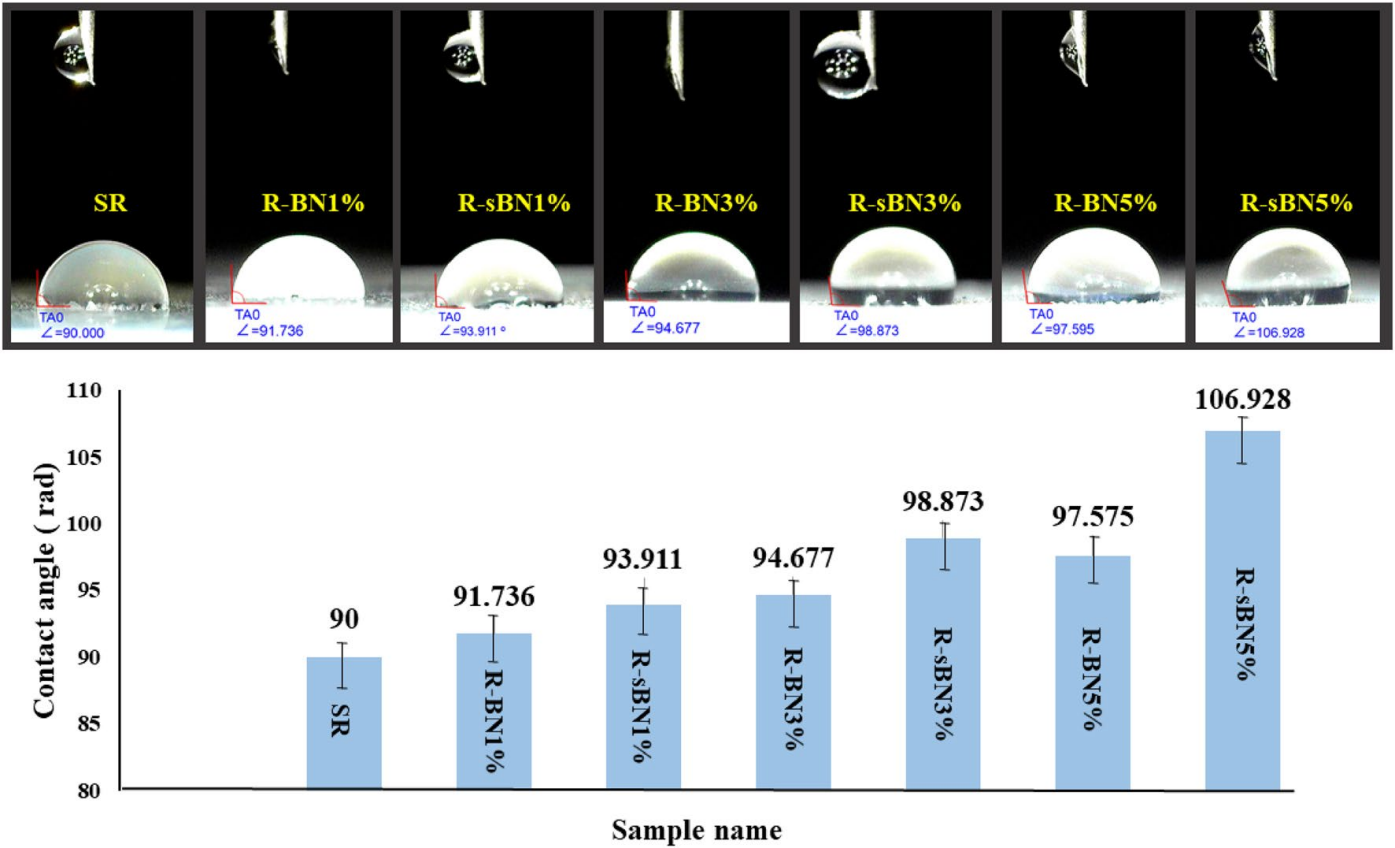
The water contact angle of SR nanocomposites.

Water Contact angle test was used to find out the effect of pure and modified h-BNs on hydrophobicity of SR which results are depicted in Fig. 11. Based on the results, incorporation of pure h-BNs increased the hydrophobicity of SR due to the lack of hydrophilic groups on h-BNs surface and chemical stability of B–N hexagonal structure that hindered water absorption. Application of higher h-BNs contents increased the surface roughness that enhanced the water contact angle.

In addition, VTMS empowered the hydrophobic nature of SR because of vinyl groups. The steric repulsion of sBNs improved the surface roughness and that is the reason for higher water contact angle of nanocomposites contained modified h-BNs. The biggest water contact angle was related to R-sBN5% (106.93°) which was 1.18 times more than that of SR (90°).

#### Mechanical properties

##### Tensile properties

**Figure 12:**
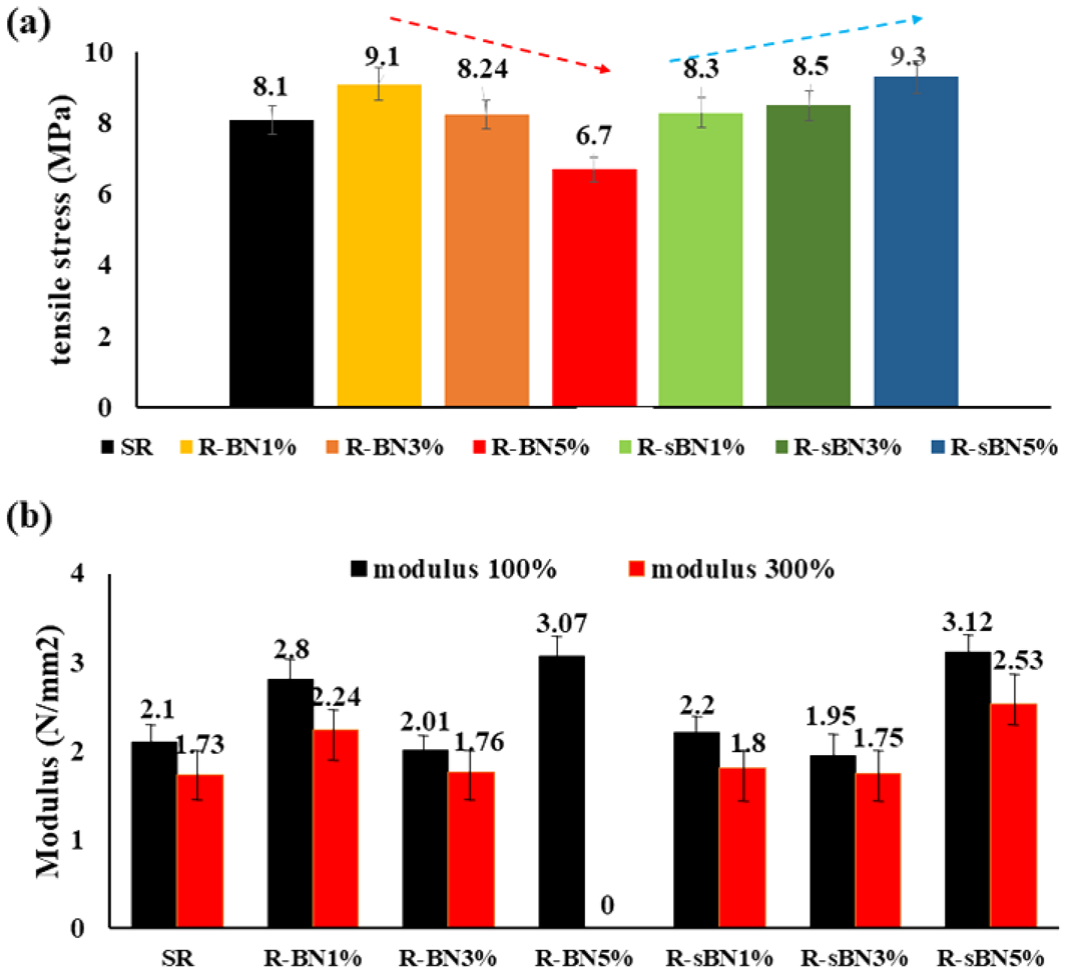
Mechanical properties of SR nanocomposites; (**a**) tensile strength, (**b**) modulus.

The tensile properties of the nanocomposites are presented in Fig. 12. In general, loading of both pure and modified h-BNs to silicon rubber increased the tensile modulus (at both 100% and 300% strains) and strength which happened mainly because of desired dispersion of h-BNs in SR matrix^28^.

Tensile strength increased for R-sBNs which became highest for R-sBN5% because of improved filler-matrix interfacial interactions^3,42^. Besides, a decline trend was observed for tensile strength of R-BNs with increasing in the filler loading content which was attributed to agglomeration of pure h-BNs in rubber matrix. After silanization, an ascending trend in tensile strength was achieved as a result of better dispersion of modified h-BNs and improved interfacial interactions to SR matrix. The modulus at 100 and 300% changed to some extent in modified nanocomposites. In R-BN5% sample, high loading content of pure h-BNs into SR matrix caused early fracture of the sample even before the strain of 300.

By incorporating pure h-BN to SR the modulus of the rubber improved that is a normal trend. However, by increasing in the h-BN content up to 3 wt%, the modulus decreased due to the agglomeration of the nano filler particles. By more using of nano filler (i.e. 5 wt%), agglomeration increased but at the same time distribution of agglomerates in the rubber matrix improved that this improved the modulus of the SR sample contained 5 wt% of h-BNs. The same trend was also seen in the samples contained modified h-BNs.

##### Thermomechanical properties

**Figure 13:**
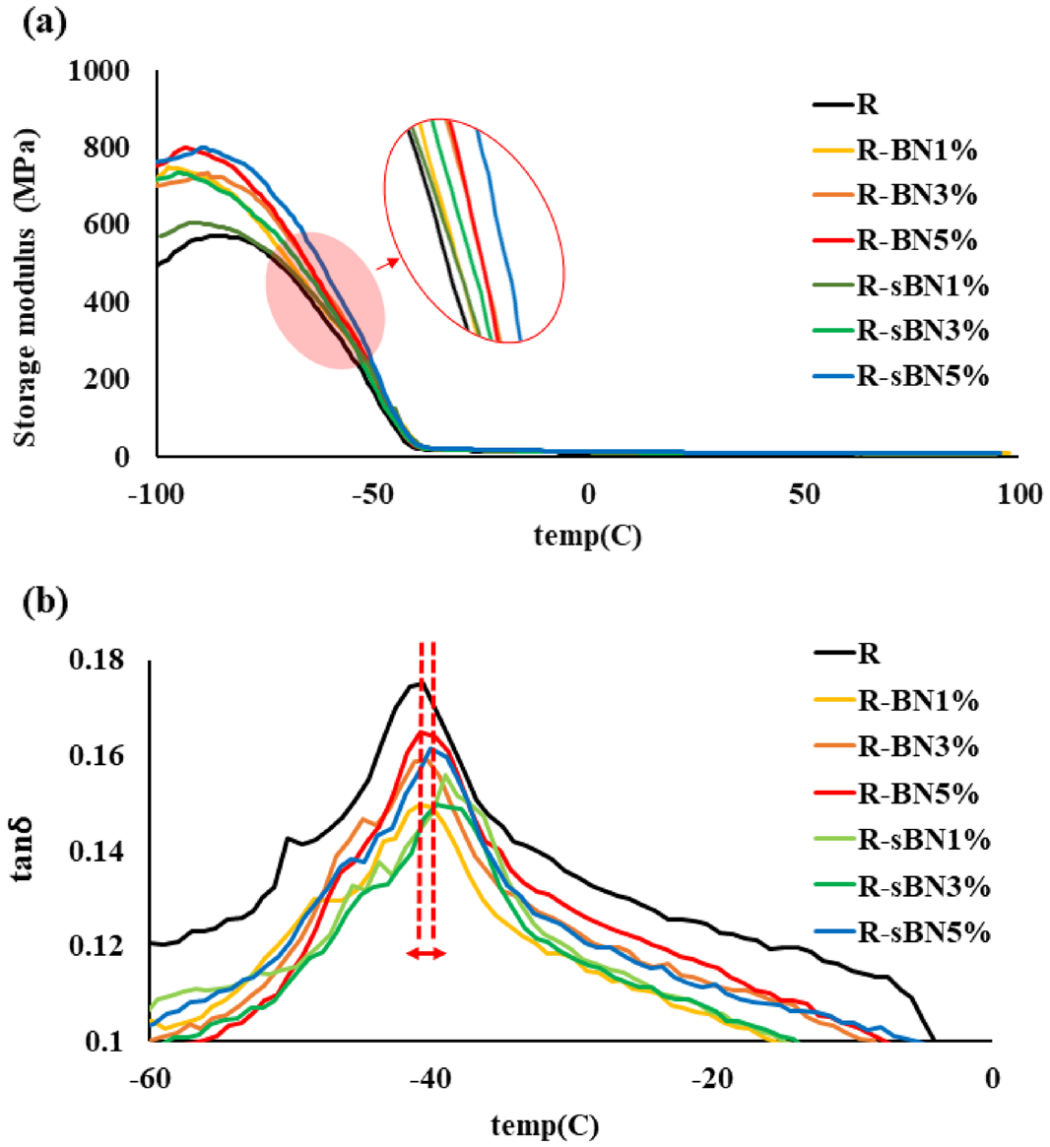
Dynamic mechanical properties of SR nanocomposites; (**a**) storage modulus and (**b**) tan δ.

DMTA analysis of the samples are shown in Fig. 13. The storage modulus is related to the sample’s elastic response. The ratio of the loss to the storage modulus gives tan δ that is called damping capacity which defines how good a material will absorb stresses through deformation. The loss modulus is also a measure of viscous behavior^43^. Based on the results, using pure and modified h-BNs improved considerably storage modulus of the silicon rubber (see Fig. 13a). The highest storage modulus was obtained at filler loading content of 5 wt% and surface modification had no effect on the storage modulus.

The glass transition temperature (Tg) of all nanocomposites were around – 40 °C but it increased about 1 °C in the case of using modified h-BNs. It was corresponded to limitations in movements of SR chains in presence of pure and modified h-BNs^32^. The tan δ of SR declined by incorporation of pure and modified h-BNs. It was corresponded to increment in the stiffness of SR in presence of h-BNs that limited movement of the chains and so decreased their damping capacity.

## Conclusions

In this work, h-BNs nanoparticles was modified by VTMS silane coupling agent which was used to improve filler–polymer interfacial interactions and dispersion of h-BNs in silicon rubber matrix. Different silane concentrations were used to optimize silane grafting ratio. The pure and modified h-BNs were characterized carefully to confirm successful grafting of silane molecules on h-BNs surface. The nanoparticles were applied at different contents to silicon rubber and the physical/mechanical/thermomechanical properties were evaluated. Based on the results the following conclusions were made:

- Functionalization of h-BNs enhanced their hydroxyl content from 0 to 2.3%.
- The silane grafting ratio increased up to 3.12% at silane concentration of 10 X.
- The crosslinking of rubber matrix (M_H_–M_L_) improved in presence of surface modified h-BNs (by 6.5% for the nanocomposite that contained 5 wt% of modified nanoparticles) compared to the pristine rubber. The pure and modified nanoparticles decreased scorch and curing times of silicone rubber, however thet modified h-BNs decreased it more.
- Thermal stability of modified nanocomposites was hindered because of lower degradation temperature of silane which is started from 400 °C and the shoulders on DTG curves of silanized samples verified the decomposition of VTMS in this temperature.
- The modified nanoparticles (i.e. at 5 wt% loading content) enhanced the water contact angle from 90° for SR to 106.93° for R-sBN5% which is about 18.1% increase.
- The tensile strength of modified nanocomposites (i.e. at 5 wt% loading content) enhanced about 14.8 and 38.8% compared to pristine SR and pure h-BN containing SR nanocomposite, respectively.
- The highest storage modulus of SR nanocomposites was obtained at 5 wt% loading content of pure and modified h-BNs which were 53.4 and 68.2% more than pristine SR, respectively. The modified h-BNs enhanced Tg (up to 1 °C) and decreased tan δ (up to 13%) because of improved interfacial interactions to SR matrix.

## Data Availability

All data generated or analyzed during this study are included in this published article.
